# Metabolic pathways regulated by γ-aminobutyric acid (GABA) contributing to heat tolerance in creeping bentgrass (*Agrostis stolonifera*)

**DOI:** 10.1038/srep30338

**Published:** 2016-07-26

**Authors:** Zhou Li, Jingjin Yu, Yan Peng, Bingru Huang

**Affiliations:** 1 Department of Grassland Science, College of Animal Science and Technology, Sichuan Agricultural University, Chengdu, Sichuan, China; 2 Department of Plant Biology and Pathology, Rurgers University, 59 Dudley Road, New Brunswick, New Jersey 08901; 3 College of Agro-Grassland Science, Nanjing Agricultural University, Nanjing, China

## Abstract

γ-Aminobutyric acid is a non-protein amino acid involved in various metabolic processes. The objectives of this study were to examine whether increased GABA could improve heat tolerance in cool-season creeping bentgrass through physiological analysis, and to determine major metabolic pathways regulated by GABA through metabolic profiling. Plants were pretreated with 0.5 mM GABA or water before exposed to non-stressed condition (21/19 °C) or heat stress (35/30 °C) in controlled growth chambers for 35 d. The growth and physiological analysis demonstrated that exogenous GABA application significantly improved heat tolerance of creeping bentgrass. Metabolic profiling found that exogenous application of GABA led to increases in accumulations of amino acids (glutamic acid, aspartic acid, alanine, threonine, serine, and valine), organic acids (aconitic acid, malic acid, succinic acid, oxalic acid, and threonic acid), sugars (sucrose, fructose, glucose, galactose, and maltose), and sugar alcohols (mannitol and myo-inositol). These findings suggest that GABA-induced heat tolerance in creeping bentgrass could involve the enhancement of photosynthesis and ascorbate-glutathione cycle, the maintenance of osmotic adjustment, and the increase in GABA shunt. The increased GABA shunt could be the supply of intermediates to feed the tricarboxylic acid cycle of respiration metabolism during a long-term heat stress, thereby maintaining metabolic homeostasis.

Heat stress induces a variety of negative effects on plant growth and development, and the damage may be further accentuated with the predictable increase of global temperature in the future^1^,^2^. It is imperative to improve heat tolerance for cool-season species growing in areas with prolonged periods of heat stress that lead to growth inhibition and even plant death^3^,^4^. Use of plant growth regulators or natural products of plants is found to be an effective approach to promote plant tolerance to various abiotic stresses, including heat stress. Recently, γ-aminobutyric acid (GABA), as an important non-protein amino acid, has been found to exhibit certain physiological functions involved in plant growth regulation and stress tolerance, such as maintenance of cytosolic pH, osmoregulation, and carbon and nitrogen metabolism^5^,^6^,^7^. Earlier studies also have demonstrated that GABA levels of plants rapidly increased when exposed to different environmental stresses^6^,^8^. Exogenous application of GABA could enhance chilling tolerance in peach fruits (*Amygdalus persica*)^9^, salt tolerance in *Nicotiana sylvestris*^10^, and drought tolerance in perennial ryegrass (*Lolium perenne*)^11^. In more recent reports, it is suggested that GABA is not just a metabolite and may act as a stress-induced intercellular signaling molecule related to the signal transduction pathway in plants^12^,^13^,^14^. However, plant adaptation to abiotic stresses is dependent on comprehensive responses associated with physiological and metabolic changes, and the role of GABA on heat stress mitigation still has not yet been fully elucidated.

Antioxidant defense, including enzymatic and non-enzymatic systems, is a fundamental detoxification system for plants to cope with oxidative damage induced by abiotic stress^15^,^16^. The ascorbate-glutathione (AsA-GSH) cycle involved in the redox reaction of AsA and GSH together with other antioxidant enzymes including superoxide dismutase (SOD), catalase (CAT), and peroxidase (POD) maintains the balance between generating and quenching of reactive oxygen species (ROS) in plant cells^17^. To some extent, antioxidant function determines the ability of plants to survive high temperature, as heat stress leads to massive ROS accumulation which damages to cell membranes and accelerates leaf senescence^18^,^19^. GABA-induced stress tolerance could be acquired by increased antioxidant ability in various plant species under different stress conditions, such as aluminium stress^20^, chilling^21^, and drought^11^,^22^. It has been reported that GABA exhibited a partial role of antioxidant protection due to increased antioxidant enzyme activity in leaves of rice (*Oryza sativa*) when exposed to short-term (10 d) high temperature (35/30 °C, day/night)^23^. However, how GABA may regulate enzymatic and non-enzymatic antioxidant pathways during a long-term heat stress has not been well documented.

Many other metabolic processes also undergo changes in plants when suffered from abiotic stress^24^. The adjustment of metabolite accumulation plays critical roles in plant adaptation to stress^25^,^26^,^27^. It has been well documented that some low-molecular-weight metabolites, such as carbohydrates and amino acids are energy source, osmotic regulants, as well as signaling molecules during stress^28^,^29^,^30^. GABA shunt is testified to the close link to the tricarboxylic acid (TCA) cycle^13^,^31^. A recent study highlighted the predominant role of GABA on metabolism, which showed GABA supply maintained amino acid metabolism and nitrogen repartition in *Arabidopsis* under carbon- or nitrogen-limiting growth conditions^32^. Although several studies have suggested that GABA supplementation to animals is beneficial to acclimate high temperature^33^,^34^,^35^, limited research has been done on GABA-induced metabolic changes for plant adaptation to prolonged periods of heat stress in cool-season plant species. The metabolic pathways that link GABA to stress-related metabolites could provide further insight into functions of GABA in regulating heat tolerance of plants.

The objectives of this study were 1) to examine whether increased GABA could improve heat tolerance in cool-season creeping bentgrass (*Agrostis stolonifera*) through physiological analysis and 2) to determine major metabolic pathways regulated by GABA that contribute to the improved heat tolerance through metabolic profiling.

## Results

### Effects of heat stress alone and with GABA on turf quality and water status

**Figure 1:**
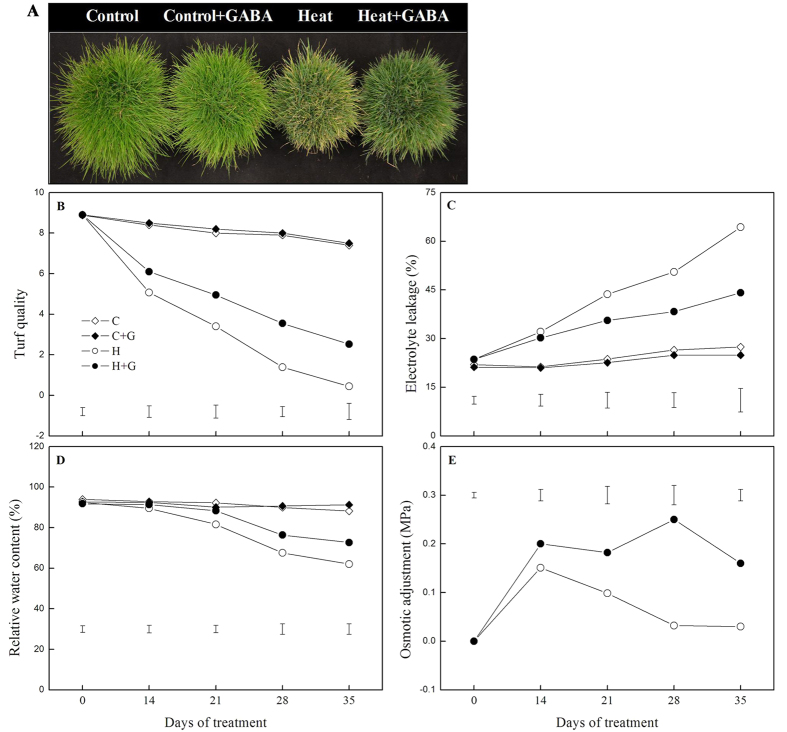
The effects of GABA on leaf water deficit and membrane stability during 35 d of 21/19 or 35/30 °C condition. (**A**) Phenotype at 35 d of treatment, (**B**) turf quality, (**C**) electrolyte leakage, (**D**) relative water content, and (**E**) osmotic adjustment. Vertical bars represent least significance difference (LSD) values at a given day of treatment (*P* = 0.05). C, control; C + G, control + GABA; H, heat; H + G, heat + GABA.

Under the non-stress condition, GABA had no significant effects on turf quality (TQ) and electrolyte leakage (EL) (Fig. 1A–C). Heat stress resulted in a significant decrease in TQ and increase in EL, but exogenous application of GABA alleviated heat-induced decline in TQ and increase in EL (Fig. 1B,C). GABA-treated plants maintained significantly higher relative water content (RWC) than untreated plants at 21, 28, and 35 d of heat stress (Fig. 1D). Leaf osmotic adjustment (OA) of both non- and GABA-treated plants increased after 14 d of heat stress and then declined in untreated plants with prolonged heat stress duration, while it was maintained at a higher level in GABA-treated plants during heat stress. GABA-treated plants showed more than 5 times higher OA than untreated plants from 28 to 35 d of heat stress (Fig. 1E).

### Effects of heat stress alone and with GABA on plant photosynthetic parameters and water use efficiency

**Figure 2:**
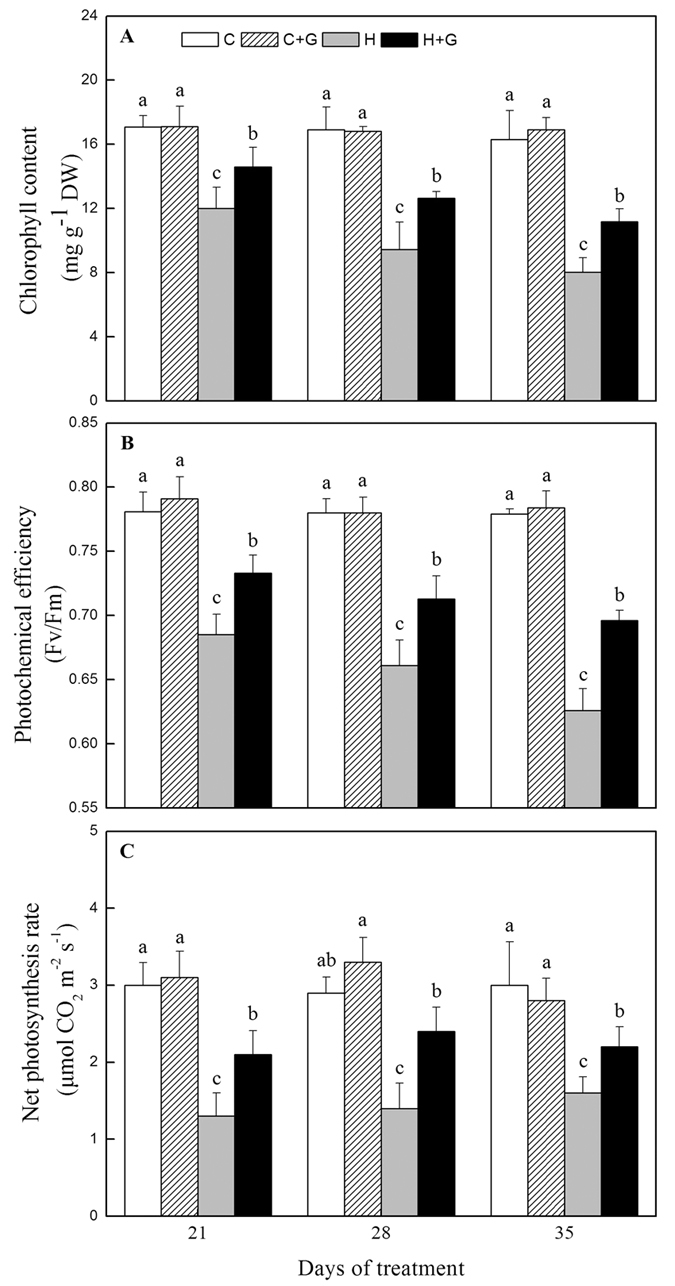
The effects of GABA on photosynthesis during 35 d of 21/19 or 35/30 °C condition. (**A**) Chlorophyll content; (**B**) Photochemical efficiency (Fv/Fm); (**C**) Net photosynthesis rate. Vertical bars above columns indicate standard error of each mean. Different letters indicate significant difference for comparison at a given day of treatment (*P* = 0.05). C, control; C + G, control + GABA; H, heat; H + G, heat + GABA.

**Figure 3:**
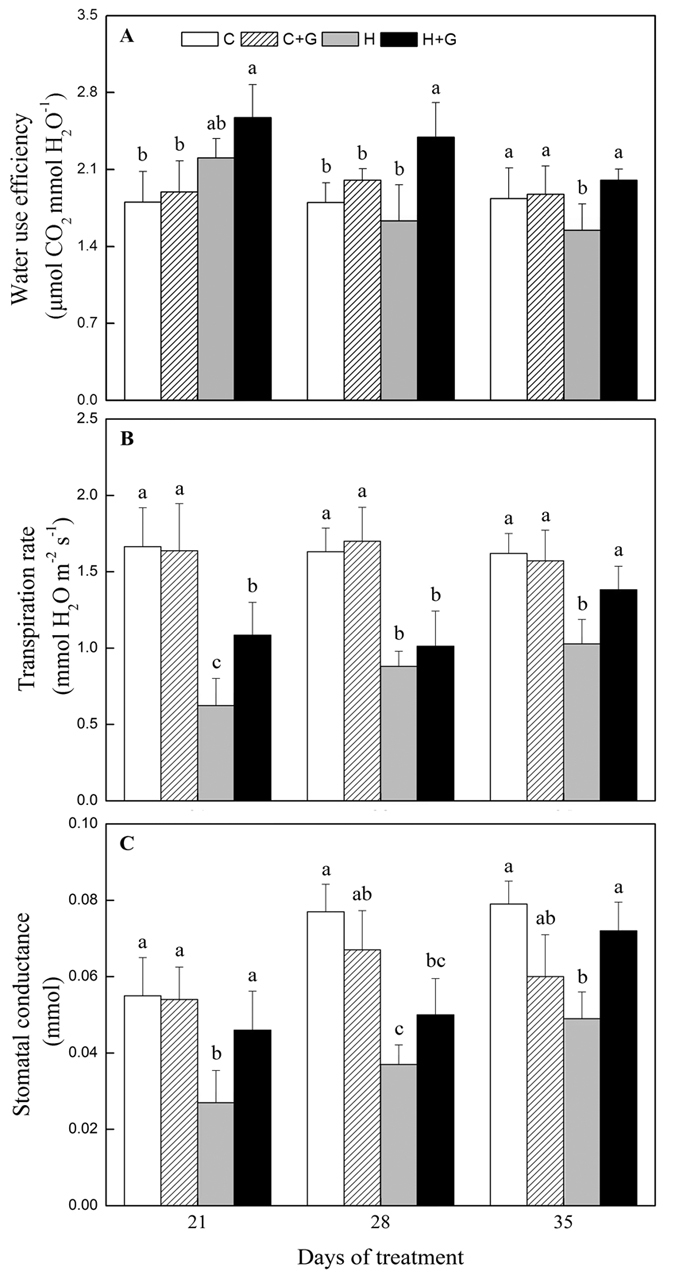
The effects of GABA on water use during 35 d of 21/19 or 35/30 °C condition. (**A**) Water use efficiency; (**B**) Transpiration rate; (**C**) Stomatal conductance. Vertical bars above columns indicate standard error of each mean. Different letters indicate significant difference for comparison at a given day of treatment (*P* = 0.05). C, control; C + G, control + GABA; H, heat; H + G, heat + GABA.

Photosynthetic parameters were not significantly affected by exogenous GABA under well-watered condition (Fig. 2A–C). Heat stress led to the reduction in chlorophyll content (Chl) content and photochemical efficiency (Fv/Fm) ratio, but with GABA application, Chl content and Fv/Fm were improved significantly from 21 to 35 d of heat stress (Fig. 2A,B). Net photosynthetic rate (Pn) declined significantly after 21 d of heat stress regardless of GABA treatment, but GABA application resulted in a 64% increase in Pn compared to untreated plants at 28 d of heat stress (Fig. 2C). Water use efficiency (WUE) calculated as the ratio of Pn/Tr was significantly higher in heat-stressed plants treated with GABA than that in heat-stressed plants without GABA at 28 and 35 d of heat stress (Fig. 3A). GABA-treated plants also maintained significantly higher transpiration rate (Tr) than untreated plants at 21 and 35 d of heat stress (Fig. 3B). As compared to non-stressed plants, heat-stressed plants without GABA application exhibited a significant reduction in stomatal conductance (gs) exposed to heat stress, while high temperature did not affect gs of these plants treated with GABA under the same stress condition (Fig. 3C).

### Effects of heat stress alone and with GABA on ROS production and antioxidant metabolism

**Figure 4:**
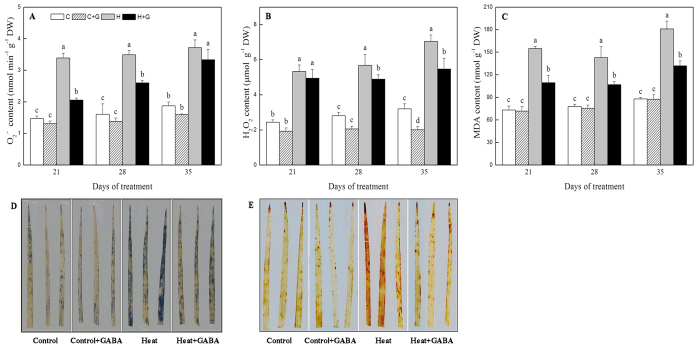
The effects of GABA on reactive oxygen species (ROS) accumulation during 35 d of 21/19 or 35/30 °C condition. (**A**) Superoxide anion radical (O_2_^·−^) content, (**B**) hydrogen peroxide (H_2_O_2_) content, (**C**) malondialdehyde (MDA) content, (**D**) O_2_^·−^ staining at 28 d of treatment, and (**E**) H_2_O_2_ staining at 35 d of treatment. Vertical bars above columns indicate standard error of each mean. Different letters indicate significant difference for comparison at a given day of treatment (*P* = 0.05). C, control; C + G, control + GABA; H, heat; H + G, heat + GABA.

The content of superoxide anion radical (O_2_^·−^), hydrogen peroxide (H_2_O_2_), and malondialdehyde (MDA) gradually increased with prolonged heat stress regardless of GABA application (Fig. 4). Foliar spray of GABA had no significant effects on O_2_^·−^, H_2_O_2_, and MDA content under non-stress condition, except at 35 d when a 34% lower H_2_O_2_ content was found in GABA-treated plants (Fig. 4A–C). Exogenous application of GABA significantly decreased heat-induced O_2_^·−^, H_2_O_2_, and MDA accumulation in leaves. O_2_^·−^ content was 39 and 26% lower in GABA-treated plants compared with non-treated plants at 21 and 28 d of heat stress. H_2_O_2_ content was 14 and 22% lower in GABA-treated plants compared with non-treated plants at 28 and 35 d of heat stress, respectively (Fig. 4A,B). From 21 to 35 d of heat stress, GABA-treated plants maintained 25% lower MDA content than untreated plants (Fig. 4C). O_2_^·−^ staining at 28 d of treatments and H_2_O_2_ staining at 35 d of treatment also showed similar results. The lower density of blue spots which indicated the accumulation of O_2_^·−^ and brown spots which indicated the accumulation of H_2_O_2_ were observed in GABA-treated leaves as compared to non-treated leaves under heat condition (Fig. 4D,E).

**Figure 5:**
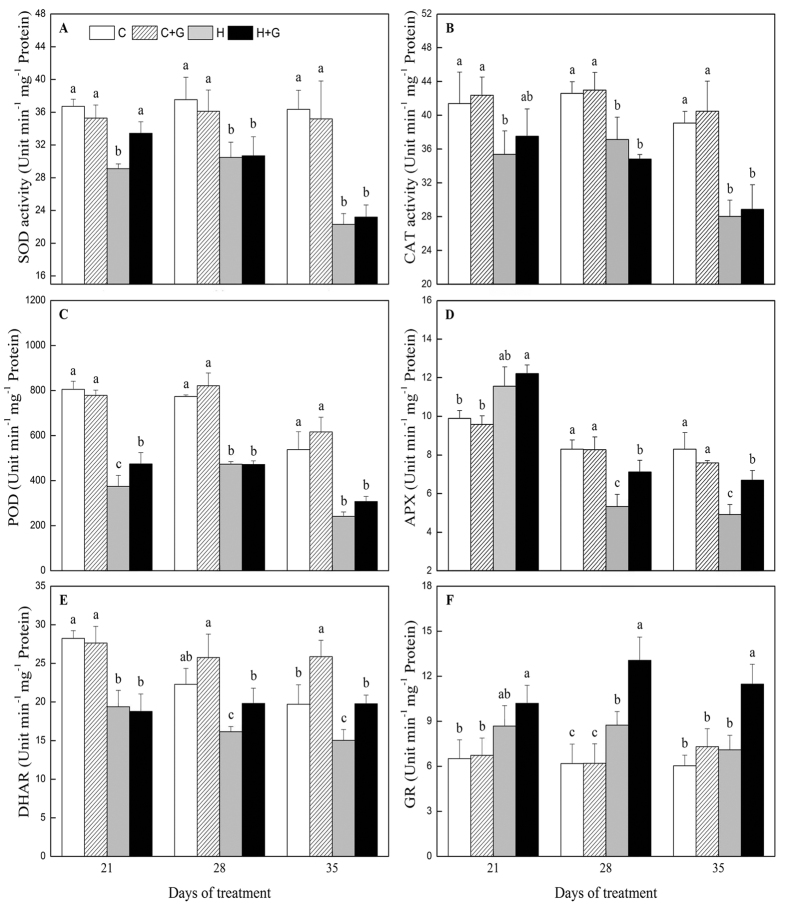
The effects of GABA on antioxidant enzyme activities during 35 d of 21/19 or 35/30 °C condition. (**A**) Superoxide dismutase (SOD) activity, (**B**) catalase (CAT) activity, (**C**) peroxide (POD) activity, and (**D**) ascorbate peroxidase (APX) activity, (**E**) dehydroascorbate reductase (DHAR) activity, (**F**) glutathione reductase (GR) activity. Vertical bars above columns indicate standard error of each mean. Different letters indicate significant difference for comparison at a given day of treatment (*P* = 0.05). C, control; C + G, control + GABA; H, heat; H + G, heat + GABA.

Heat stress caused decreases in SOD, CAT, and POD activities and an increase in APX activity. The activities of SOD, CAT, POD, and APX were not affected by exogenous GABA under non-stress condition (Fig. 5A–D). As demonstrated in Fig. 5B, GABA application also had no significant effect on CAT activity in heat-stressed plants. With application of GABA, plants showed 15 and 27% higher SOD and POD activity at 21 d of heat stress, respectively (Fig. 5A,C). Similarly, ascorbate peroxidase (APX) activity increased by 33 and 36% in GABA-treated plants than that in untreated plants at 28 and 35 d of heat stress, respectively (Fig. 5D). Dehydroascorbate reductase (DHAR) activity was enhanced in GABA-treated plants compared to untreated plants both under non-stressed and heat stress condition (Fig. 5E). GABA application had no significant effects on glutathione reductase (GR) activity under non-stressed condition, but GABA-treated plants showed 49 and 62% increases in GR activity relative to untreated plants when exposed to 28 and 35 d of heat stress (Fig. 5F).

**Figure 6:**
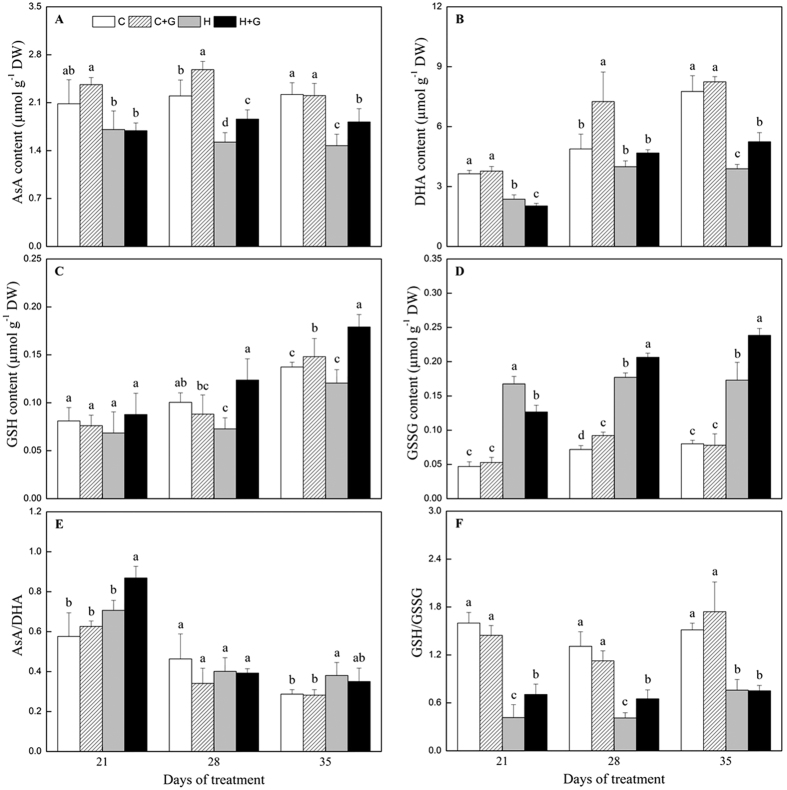
The effects of GABA on non–enzymatic antioxidants during 35 d of 21/19 or 35/30°C condition. (**A**) reduced ascorbate (AsA), (**B**) dehydroascorbic acid (DHA), (**C**) reduced glutathione (GSH), (**D**) oxidized glutathione (GSSG), (**E**) AsA/DHA ratio, and (**F**) GSH/GSSG ratio. Vertical bars above columns indicate standard error of each mean. Different letters indicate significant difference for comparison at a given day of treatment (*P* = 0.05). C, control; C + G, control + GABA; H, heat; H + G, heat + GABA.

Heat stress reduced the content of ascorbic acid (AsA) and dehydroascorbic acid (DHA). However, with the application of GABA, plants showed significantly higher AsA content under both non-stress and heat stress conditions (Fig. 6A,B). In non-stressed plants treated with GABA, AsA and DHA content went up by 17 and 49%, respectively, over those plants without GABA at 28 d, and heat-stressed plants treated with GABA had 24 and 35% higher AsA and DHA content than heat alone at the last day of heat stress, respectively (Fig. 6A,B). In response to heat stress, reduced glutathione (GSH) and oxidized glutathione (GSSG) content gradually increased in leaves of creeping bentgrass (Fig. 6C,D). GSH and GSSG were found to increase by 1.4-fold in GABA-treated plants compared to non-treated plants at 35 d of heat stress (Fig. 6C,D). The AsA/GSH and GSH/GSSG ratio were not significantly different between non-treated and GABA-treated plants under non-stress condition, while heat-stressed plants treated with GABA exhibited significantly higher AsA/GSH at 21 d and GSH/GSSG at 21 and 28 d than heat-stressed plants without the application of GABA (Fig. 6E,F).

### Metabolic profiling of metabolite changes in response to heat stress alone and with GABA

More than 300 peaks were detected by Gas Chromatography-Mass Spectrometer (GC-MS) and over 250 putative metabolites were found in leaves of creeping bentgrass. A total of 57 metabolites differentially accumulated in response to at 35 d of heat stress or with GABA treatment were identified and quantified in leaves of creeping bentgrass, mainly including 10 amino acids, 22 organic acids, 17 sugars, and 8 sugar alcohols, and the basic information of identified metabolites is presented in Table 1.

**Figure 7:**
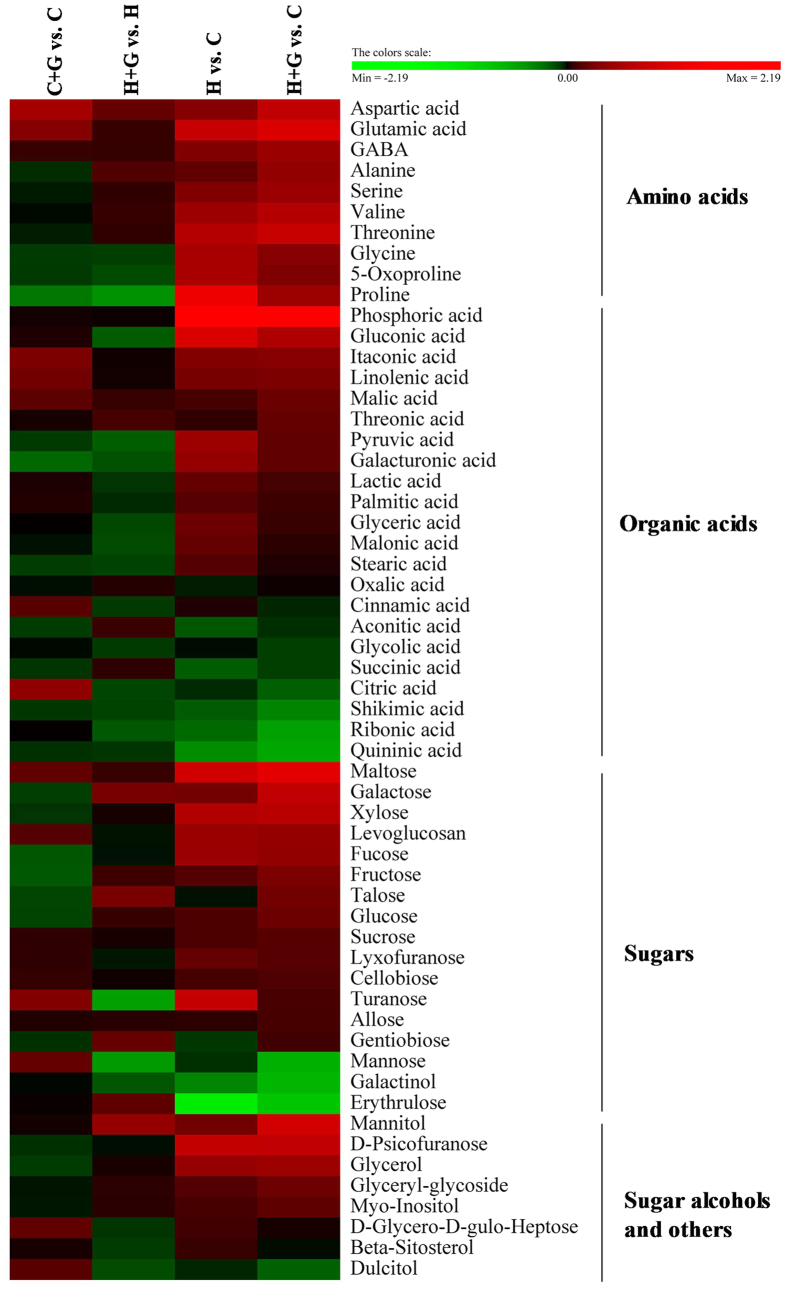
Heat map of changes in 57 metabolites levels in creeping bentgrass at 35 d in response to exogenous GABA application and heat stress. The log_2_ fold change ratios are shown in the results. Red indicates an up–regulation, and green indicates a down–regulation. C + G vs. C and H + G vs. H implied the effects of exogenous GABA on metabolites under control or heat condition, respectively. H vs. C and H + G vs. C implied the effects of heat stress on metabolites without or with GABA application, respectively. C, control; C + G, control + GABA; H, heat; H + G, heat + GABA.

**Figure 8:**
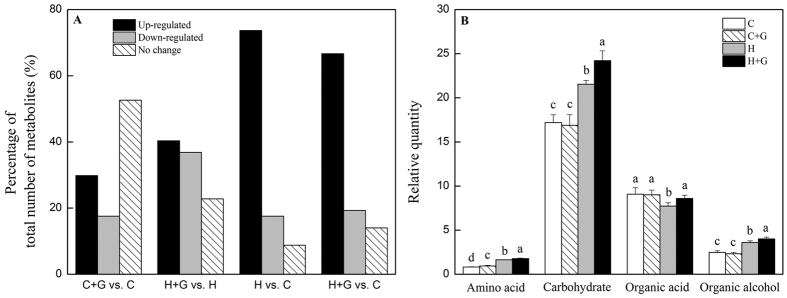
Change to (**A**) the percentage of total number of metabolites (%), and (**B**) total relative amino acid, organic acid, sugar and sugar alcohol content at 35 d of treatment. Vertical bars above columns indicate standard error of each mean. Different letters indicate significant difference (*P* = 0.05). C, control; C + G, control + GABA; H, heat; H + G, heat + GABA.

Heat map showed changes in the levels of 57 metabolites in response to exogenous application of GABA and heat stress (Fig. 7). In order to analyze metabolic pathways regulated by GABA and heat stress, the effects of GABA were investigated by comparing GABA treatment with untreated control under non-stress condition (C + G vs. C) and under heat stress (H + G vs. H), and metabolites were affected by heat stress through comparing heat stress treatments without or with GABA application with non-stress control (H vs. C and H + G vs. C). GABA application led to increases in the abundance of 17 metabolites and 23 metabolites, and decreases in the abundance of 10 metabolites and 21 metabolites under non-stress and heat stress, respectively. For both untreated and GABA-treated plants, heat stress led to increases in 23 metabolites and decreases in 3 metabolites (Fig. 8A). As shown in Fig. 8B, the total content of amino acids, carbohydrates, and organic alcohols significantly increased both in untreated and GABA-treated plants under heat stress, while total content of organic acids decreased under heat stress in GABA untreated plants, but increased in GABA-treated plants. GABA-treated plants exhibited 9, 12, 11, and 11% higher content of amino acids, carbohydrates, organic acids, and organic alcohols, respectively, than untreated plants under heat stress. Exogenous application of GABA had no significant effects on total content of carbohydrates, organic acids, and organic alcohols content, but enhanced accumulation of amino acids under non-stress conditions (Fig. 8B).

**Figure 9:**
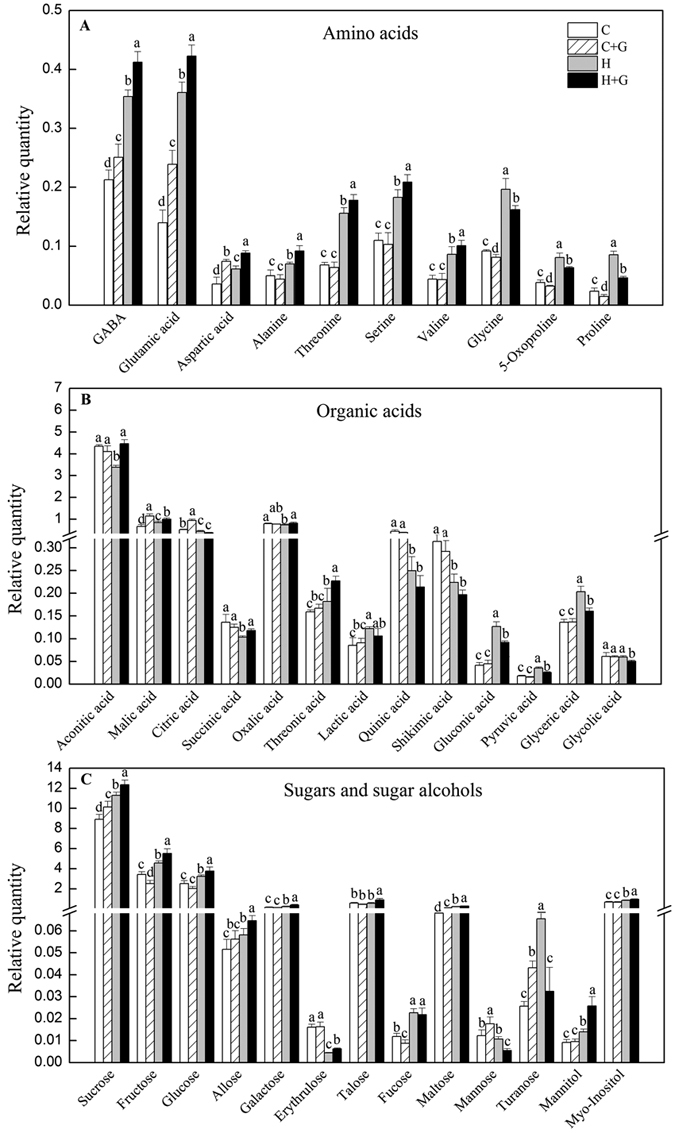
Change to (**A**) relative amino acids, (**B**) organic acids, and (**C**) sugars and sugar alcohols content at 35 d of treatment. Vertical bars above columns indicate standard error of each mean. Different letters indicate significant difference (*P* = 0.05). C, control; C + G, control + GABA; H, heat; H + G, heat + GABA.

The levels of key metabolites associated with heat tolerance and GABA were showed in Fig. 9. Heat stress caused significantly increases in 10 amino acids, and foliar application of GABA further improved the accumulation of 7 amino acids (GABA, glutamic acid, aspartic acid, alanine, threonine, serine, and valine) under heat stress. However, GABA-treated plants had significantly lower glycine, 5-oxoproline, and proline content than untreated plants under non-stress and heat stress conditions (Fig. 9A). For changes of organic acids, GABA-treated plants exhibited significantly higher aconitic, malic, citric, succinic, oxalic, and threonic acid under non-stress or heat condition and no significant differences in lactic, quinic, and shikimic acid were observed between GABA-treated and untreated plants under both temperature conditions, while gluconic, pyruvic, glyceric, and glycolic acid were down-regulated in GABA-treated plants compared to untreated plants under heat stress (Fig. 9B). The accumulation of 8 sugars (sucrose, fructose, glucose, allose, galactose, erythrulose, talose, and maltose) was enhanced by GABA application under heat stress except fucose, mannose, and turanose. In addition, exogenous GABA also resulted in significant increases in two sugar alcohols, mannitol and myo-inositol, under heat stress (Fig. 9C).

### Metabolic pathways affected by GABA and heat stress

**Figure 10:**
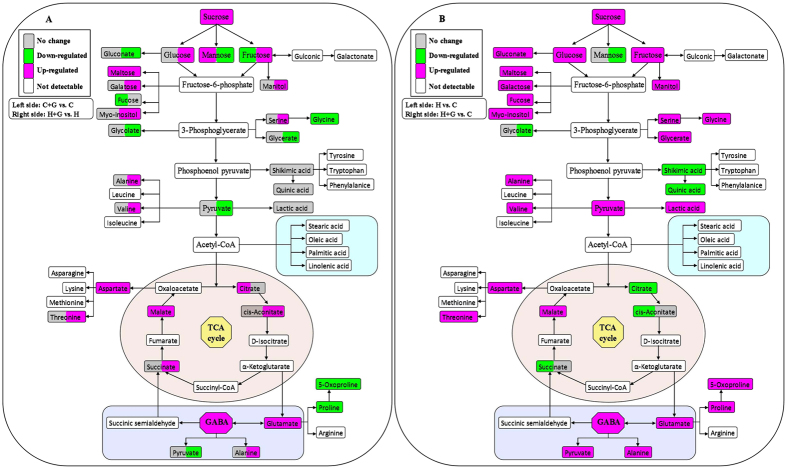
Assignment of the 30 metabolites from 57 assayed metabolites to the metabolic pathway. (**A**) The effects of increased GABA on metabolites under control or heat condition, and the color of left side each box means C + G vs. C, and the color of right side each box means H + G vs. H. (**B**) The effects of heat stress on metabolites without or with GABA application, and the color of left side each box means H vs. C, and the color of right side each box means H + G vs. C. C, control; C + G, control + GABA; H, heat; H + G, heat + GABA.

**Figure 11:**
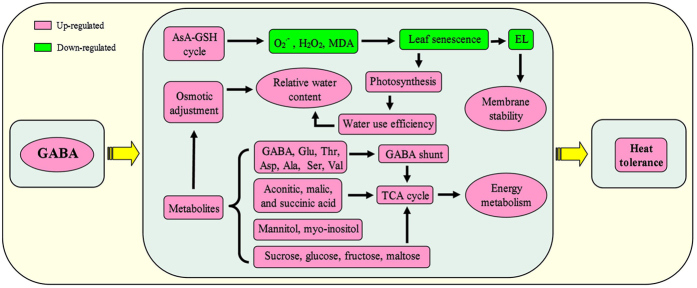
A proposed model for GABA–induced heat tolerance in creeping bentgrass associated with physiological and metabolic changes. Red indicates an up–regulation, green indicates a down–regulation.

Figure 10 presented central pathways of biosynthetic metabolism involved in the GABA shunt, the TCA cycle and the primary metabolism. Out of 57 identified metabolites, 30 (10 amino acids, 11 organic acids, and 9 sugars and sugar alcohols) were assigned to these metabolic pathways. Increased endogenous GABA content induced by foliar application of GABA enhanced GABA shunt and TCA cycle both under non-stress control (C + G vs. C) and heat (H + G vs. H) conditions, while it resulted in a decrease in proline content (Fig. 10A). Under heat stress, GABA application caused increases in endogenous GABA content and the accumulation of glucose, fructose, maltose, sucrose, and most of amino acids. In contrast, gluconate, glycolate, glycerate, and pyruvate content declined (Fig. 10A). Figure 10B showed the effects of heat stress on metabolic pathways without (H vs. C) or with GABA application (H + G vs. C) as compared to non-stress control. Succinate and cis-aconitate decreased only in heat-stressed plants without GABA, but not in heat-stressed plants with GABA application, while mannose and glycolate only declined in heat-stressed plants treated with GABA (Fig. 10B). To summarize the results, a proposed model for GABA-induced heat tolerance in creeping bentgrass associated with physiological and metabolic changes was presented in Fig. 11. Increased endogenous GABA improved AsA-GAH cycle to decrease ROS (O_2_^·−^ and H_2_O_2_) and MDA content associated with delaying leaf senescence and maintaining cell membrane stability. The enhancement of OA together with significantly increased WUE could contribute to the maintenance of RWC under heat condition. In addition, GABA-induced the accumulation of metabolites was involved in GABA shunt, amino acids, and sugar metabolism, which could be the supply of intermediates to feed the TCA cycle of respiration metabolism during a long-term heat stress, thereby maintaining metabolic homeostasis (Fig. 11).

## Discussion

Heat stress damages include accelerated leaf senescence associated with loss of chlorophyll and membrane stability and inhibition of growth and photosynthesis, as well as the induction of oxidative stress, as found in creeping bentgrass in this study and other plants species^36^,^37^,^38^. Our current findings showed that exogenous application of GABA effectively alleviated these heat stress damages in creeping bentgrass. Delayed leaf senescence (increased Chl content and Fv/Fm) could contribute to the increased Pn due to GABA application in leaves of creeping bentgrass. Similarly, increases in Chl content and photosynthesis by GABA application have been observed in other plant species exposed to hypoxia stress^39^. Heat stress also may cause water deficit in plants due to imbalanced water loss and water uptake^40^,^41^. Plants treated with GABA also exhibited improvement in OA and retained higher RWC, supporting the role of GABA in regulating plant water relations. In addition, higher transpiration was in conjunction with greater Pn leading to significant improved WUE in GABA-treated plants during heat stress. The physiological analysis suggested that GABA could alleviate heat damages in photosynthesis by suppressing leaf senescence and balancing water relations through improving osmotic adjustment and WUE.

GABA-induced stress tolerance could also involve changes in antioxidant enzyme activities associated with mitigation of oxidative injury^20^,^21^,^42^. Nayyar *et al*.^23^ reported that exogenous GABA resulted in increases in SOD, CAT, APX, and GR activities in rice seedlings after a relative short-term (10 d) heat stress (35/30 °C, day/night), indicating its protective role in scavenging heat-induced ROS. In this study, under prolonged periods (28 d) of heat stress (35/30 °C, day/night), GABA enhanced the activity of several key enzymes involved in AsA-GSH cycle and the accumulation of nonenzymatic antioxidants (AsA and GSH) while it had no significant effects on activating SOD, CAT and POD. As a result, ROS level and lipid peroxidation were found to decline notably in GABA-treated plants under heat stress. Our results suggested that the ability of GABA-treated plants to alleviate oxidative damages might be mainly the result of activation of both enzymatic and nonenzymatic metabolism in the AsA-GSH cycle during prolonged periods of heat stress, which along with the alleviation of heat-damages in photosynthesis and enhancement of efficient water relations, as well the maintenance of osmotic adjustment, conferring GABA-induced heat tolerance in creeping bentgrass.

The maintenance of metabolic balance and the accumulation of specific metabolites associated with increased endogenous GABA could be associated with the positive effects of GABA on heat tolerance. Carbohydrates are among the most abundant metabolites in plants, which play essential roles in plant tolerance to abiotic stresses, such as serving as energy source, osmoregulants, and signaling molecules^43^,^44^. Sugar alcohols, such as mannitol and myo-inositol, exhibit roles in signal transduction and scavengers of ROS against abiotic stress^45^,^46^,^47^. Increased carbohydrate accumulation has been associated with enhanced heat tolerance^48^,^49^. GABA application resulted in significant increases in the content of most of sugars identified in this study as well as sugar alcohols, implying the GABA-induced accumulation of carbohydrates could be related to enhanced Pn and osmotic adjustment among various other critical functions imparting heat tolerance.

Amino acids are another important primary metabolites in plants, which also serve as osmolytes as well as precursors for synthesis of proteins and secondary metabolites for stress defense^50^. Jespersen *et al*.^51^ found that exogenous nitrogen stimulated the accumulation of 6 amino acids in leaves of creeping bentgrass associated with enhanced heat tolerance. In the current study, the application of exogenous GABA resulted in increases of 7 amino acids (GABA, glutamic acid, aspartic acid, threonine, serine, alanine, and valine) of total 10 identified amino acids, suggesting the supply of GABA could be critically important for amino acid metabolism under heat conditions. GABA and glutamic acid which can transform from each other directly both serve as nitrogen resource^13^,^32^. Furthermore, glutamic acid was also involved in chlorophyll biosynthesis^52^,^53^. Increased aspartic acid, as an important precursor for many other amino acids, has been found to link to nitrogen metabolism and delayed leaf senescence under heat stress^48^,^51^,^54^. Threonine synthesis and catabolism could maintain isoleucine needs of plants, which had key functions on regulation of jasmonic acid signaling and heat shock response^55^,^56^,^57^. Melatonin-improved multiple abiotic stresses tolerance and CaCl_2_-induced cold tolerance both were associated with significantly up-regulated alanine and valine in bermudagrass (*Cynodon dactylon*) through the analysis of metabolome^58^,^59^. *Arabidopsis* mutant *shmt1* with a loss-of-function serine hydroxymetyltransferase was more susceptible to high light intensity and salt stress than wild type implying the importance of serine accumulation on stress tolerance^60^. Thus, all of the accumulation of these amino acids could contribute to GABA-induced heat tolerance via involvement in the supply of metabolic adjustment, antioxidants, OA, and other stress defense functions.

It has been well documented that glycine is the precursor of glutathione synthesis^61^. Interestingly, glycine, 5-oxoproline, and proline declined with GABA application under heat stress. Thus, significant down-regulated glycine content could be due to be transferred to synthesis of glutathione for antioxidant defense, since significantly increased glutathione was observed in GABA-treated plants as compared to untreated plants under heat stress. Proline is known as a stress-related amino acid, and its level varies with the severity of stress and the level of stress tolerance of plant species^62^,^63^. Excess accumulation of proline could attenuate heat tolerance in *Arabidopsis* seedlings^64^. The study of Du *et al*.^48^ implied that higher increases in proline content reflected the greater damages in bermudagrass and Kentucky bluegrass during early period of heat stress. The lower proline content of GABA-treated plants could reflect lesser heat-induced damages in creeping bentgrass. Additionally, based on the analysis of metabolic pathways, increased GABA shunt could be the supply of pyruvate and succinic semialdehyde to feed the TCA cycle instead of going to proline metabolism.

The multiplicity of organic acids result in the complexity of their functions involved in regulation of pH, stress defense, energy metabolism and detoxifcation in response to different environmental stresses^65^,^66^. Heat-caused decrease in total organic acids content was observed in GABA-untreated creeping bentgrass, while GABA-treated plants could maintain organic acids at a normal level. The result suggested that improved GABA alleviated heat-induced the deficiency of organic acids. Many organic acids are involved in TCA cycle which is most critical process of energy metabolism to drive ATP synthesis and biosynthesis in plants^67^. Generally, heat stress enhanced respiration to activate energy metabolism which may deplete intermediates of TCA cycle pool^49^,^68^,^69^. Increased GABA metabolism may stimulate organic metabolism in the TCA cycle of respiration, such as aconitate, succinate, and malate, since these metabolites were maintained to a greater extent in GABA-treated plants as compared to that in untreated plants under heat stress. In addition, oxalic acid played an important role in low-temperature response because of the increase in antioxidant potential in pomegranate (*Punica granatum*) and mango (*Mangifera indica*)^70^,^71^. Exogenous oxalic acid alleviated heat stress in alfalfa (*Medicago sativa*) through activation of antioxidant enzymes^72^. The finding of Merewitz *et al*.^73^ indicated that cytokinin-regulated drought tolerance could be correlated with an accumulation of oxalic acid in creeping bentgrass. Increased oxalic acid induced by GABA was observed in our study, suggesting GABA-regulated heat tolerance may also involve oxalic acid accumulation.

In summary, physiological and metabolic analyses of plants treated with GABA under non-stress and heat stress conditions demonstrated that GABA could improve heat tolerance through regulating multiple physiological processes and metabolic pathways, including the improvement of antioxidant metabolism, inhibition of leaf senescence, balance of photosynthesis and transpiration, and enhancement of osmotic adjustment, as well as the accumulation of amino acids, carbohydrates, organic acids and alcohols. The enhanced carbohydrate metabolism, amino biosynthesis, GABA shunt and TCA cycle could play important roles for GABA-induced heat tolerance. These results highlight the beneficial roles of GABA in heat responses. Future research will focus on the role of GABA as stress signaling molecules on differential expression of genes and proteins controlling those aforementioned metabolic pathways.

## Methods

### Plant material and treatment

Creeping bentgrass (cv. Penncross) sods were collected from Horticultural Farm II at Rutgers University, North Brunswick, NJ. The sod pieces were planted in polyvinyl chloride tubes (30 cm length, and 10 cm diameter) filled with fritted clay in a greenhouse for two months (September–October, 2014). The greenhouse had average day/night temperatures of 23/16 °C and 790 mmol m^−2^ s^−1^ photosynthetically active radiation (PAR) with natural sunlight and supplemental sodium lights when lack of natural sunlight. Plants were fertilized weekly with half-strength Hoagland’s nutrient solution^74^ and trimmed twice a week to maintain a canopy height of approximately 4 cm. Plants were transferred to controlled growth chambers after 2-month establishment in the greenhouse. The growth chambers were controlled at day/night temperatures of 21/19 °C, 70% relative humidity, and 12-h photoperiod at PAR of 660 mmol m^−2^ s^−1^ at the canopy level. Plants were maintained in those conditions for 7 d to allow plant acclimation to the growth chamber conditions prior to the imposition of heat treatment.

For the GABA treatment, all plants were sprayed three times at two-day intervals before exposed to heat stress with equal volume of 0.5 mM GABA solution or water (non-GABA treated control), and the duration of pretreatment was 5 d. The concentration of GABA was chosen based on a preliminary test with a range of concentrations (0, 0.5, 1, 2, 4 mM) for the most effective concentration on phenotypic changes. GABA-treated plants or non GABA-treated control plants were then subjected to the following four treatments in growth chambers for 35 d: 1) Non-stress control: plants were irrigated every two days to maintain soil water content at the pot capacity and maintained at 21/19 °C (day/night); 2) Non-stress control treated with GABA; 3) Heat stress: plants were exposed to 35/30 °C (day/night) conditions; and 4) Heat stress treated with GABA. Thus, the experiment had four treatments, and each treatment had four replicates. The experiment design was a split-plot design with temperature as the main plot and GABA treatments as the sub-plot. Each temperature treatment (21/19 °C or 35/30 °C, day/night) was replicated in four growth chambers (a total of eight chambers), and one pot (replicate) of GABA-treated and non-treated plants were placed in each growth chambers set at 21/19 °C or 35/30 °C.

### Physiological analysis

TQ was evaluated based on color, density, and uniformity of the grass using a scale of 1 to 9; 9 being fully turgid, dense green canopy, 6 being minimal acceptable level, and 1 being completely desiccated and brown plants^75^. Leaf EL was calculated as the percentage of C_initial_/C_max_^76^. After washing three times with deionized water, 0.1 g fresh leaves were immersed in 35 mL of deionized water and shaken for 24 h to measure initial conductivity (C_initial_) using a conductivity meter (YSI Model 32, Yellow Spring, OH). Leaves then were autoclaved at 120 °C for 20 min and the conductance of the solution was measured as maximum conductance (C_max_). Leaf RWC was calculated using the formula RWC (%) = [(FW–DW)/(TW–DW)] × 100^77^. Fresh leaves were collected form plants and immediately weighed for fresh weight (FW), and then leaves were immerged in distilled water for 12 h at 4 °C. Turgid leaves were gently wiped dry and weighed for turgid weight (TW). Leaves then dried at 80 °C for 72 h to get a dry weight (DW). For determination of OA, fresh leaf tissues were submerged in deionized water for 8 h at 4 °C to fully hydrate leaves. Tissues were blotted dry and frozen in liquid nitrogen for further analysis. Following thawing in ice bath, leaves were ground with a micropestle to extract leaf sap and 10 mL sap was inserted into an osmometer (Wescor, Inc., Logan, UT) to determine osmolality (mmol kg^−1^). According to the formula osmotic potential = ([osmolality] [0.001][2.58]), osmolality was converted to osmotic potential. OA was then calculated as the difference in osmotic potential at full turgor between stressed leaves and well-watered control leaves^78^.

For leaf Chl content analysis, fresh leaves (0.1 g) were immerged in 10 mL of dimethyl sulphoxide in the dark for 48 h, and then the leaf extract was measured at 663 nm and 645 nm with a spectrophotometer (Spectronic in Instruments, Rochester, NY, USA). The formula described in Arnon^79^ was used for calculating chlorophyll content. For measurement of Fv/Fm, A layer of leaves were adapted to darkness for 30 min using leaf clips, and the Fv/Fm ratio was recorded with the fluorescence meter (Fim 1500; Dynamax, Houston, TX, USA). Three measurements of Fv/Fm ratio were taken per replicate at each sampling day. Pn, Tr, and gs of 10 individual leaves per replicate per treatment were measured using a photosynthetic apparatus (Li-Cor 6400, Li-Cor, Inc., Lincoln, NE). WUE was calculated following the formula: WUE = Pn/Tr. For the above measurements, leaves were placed in the leaf chamber, which provided 400 μl L^−1^ CO_2_ and 800 μmol photon m^−2^ red and blue light. Leaf samples were then cut from plants and scanned with Magic Wand^TM^ Portable Scanner (PDS-ST415-VPS, VuPoint Solutions) to measure leaf area which was used to calculate Pn, Tr, and gs.

### The measurement of ROS and MDA content

For the measurement of generation of O_2_^·−^, 0.1 g leaves were ground with 1.5 ml 65 mM PBS (pH 7.8) and then centrifuged at 10000 g for 30 min at 4 °C. The supernatant was collected. The reaction mixture containing supernatant (0.5 ml), PBS (0.5 ml), and 10 mM hydrochloride (0.1 ml) was incubated at water bath (25 °C) for 20 min. A 2 ml mixed solution (58 mM sulfanilamide and 7 mM α-naphthylamine) was added into reaction mixture for another 20 min at 25 °C water bath and then the reaction was extracted with 2 ml chloroform and the absorbance was measured at 530 nm^80^. The H_2_O_2_ was assayed according to potassium iodide method^81^. Briefly, 0.1 g leaves were homogenized with 5 ml 0.1% TCA and centrifuged at 12000 g for 20 min. 0.5 ml 10 mM potassium phosphate and 1 ml 1 M KI were added to 0.5 ml of supernatant. The absorbancy of reaction was recorded at 390 nm. To analyze MDA, 0.2 g leaves was ground on ice with 2 ml of 50 mM cold phosphate buffer saline (PBS) (pH 7.8). The homogenate was centrifuged at 12000 g for 30 min at 4 °C. A 0.5 ml supernatant and 1.0 ml reaction solution (20% w/v trichloroacetic acid and 0.5% w/v thiobarbituric acid) were added to the pellet and incubated at 95 °C for 15 min, and then cooled quickly in cool water. The reaction solution was centrifuged at 8000 g for 10 min. The absorbance of the reaction solution was measured at 532 and 600 nm. The concentration of MDA was calculated according to the formula described by Dhindsa *et al*.^82^. For determination of O_2_^·−^ or H_2_O_2_ staining, leaves were stained with 2 mM nitrobluetetrozolium (NBT) in 20 mM PBS (pH 6.8) for 12 h or 0.1% (w/v) 3-diaminobenzinidine (DAB, pH 3.8) for 24 h, respectively, and then decolorated with ethanol and rinsed with deionized water^83^,^84^.

### The analysis of antioxidant enzyme activity and content of non-enzymatic antioxidants

The supernatant, which was collected from MDA extraction, was further used for the assay of antioxidant enzyme activity. For SOD activity, 0.05 ml enzyme extract was mixed with 1.45 ml reaction solution (1.1 ml of 50 mM PBS, 100 μ of 195 mM methionine, 150 μl of 60 μM riboflavin, and 100 μl of 1.125 mM NBT), and the change of absorbance was recorded at 560 nm^85^. The activities of POD and CAT were determined by using the methods of Chance and Maehly^86^. For the assay of POD, 0.05 ml H_2_O_2_ (0.75%), 0.5 ml guaiacol solution (0.25%) and 0.995 ml PBS (100 mM, pH 5.0) were added in 0.05 ml enzyme extract, and then the mixture was gently shaken. For the assay of CAT, 0.5 ml H_2_O_2_ (45 mM) and 1 ml PBS (50 mM, pH 7.0) were mixed with 0.05 ml enzyme extract. The absorbance changes of reaction solution were monitored at 460 or 240 nm every 10 seconds for 6 times for POD or CAT, respectively. For measurement of APX activity, 0.05 ml of enzyme extract was added into 1.5 ml of reaction solution containing 10 mM AsA, 5 mM H_2_O_2_, 0.003 mM EDTA, and 100 mM PBS (pH 5.8) and the change of absorbance was recorded every 10 seconds for 6 times at 290 nm^87^. DHAR and GR activities were determined according to the method of Cakmak *et al*.^88^ and the changes in absorbance were monitored at 340 nm for GR or 265 nm for DHAR every 10 seconds for 1 min. Protein content was determined using Bradford’s^89^ method.

AsA, DHA, GSH and GSSG content were measured using the method of Gossett *et al*.^90^. After homogenization of 0.2 g of leaves in 3 ml 5% phosphoric acid, the sample was centrifuged at 12000 g for 15 min. Total ascorbate was determined in a reaction mixture consisting of 100 μl supernatant, 250 μl 150 mM KPO_4_ buffer (pH 7.4) containing 5 mM EDTA and 50 μl 10 mM DTT. AsA was measured in a similar reaction mixture but the DTT was replaced by an equal amount of H_2_O. The further reaction was developed in both mixtures above by the addition of the following reagents: 200 μl 10% TCA, 200 μl 44% phosphoric acid, 200 μl α, α′-dipyridyl, and 100 μl FeCl_3_ and the mixtures were incubated at 40 °C for 40 min. The absorbency of reaction solution was read at 525 nm. The concentration of DHA was estimated from the difference of total ascorbate and AsA. For determination of glutathione, 0.2 g leaf tissues were ground in 3 ml of 6% phosphoric acid (pH 2.8). The homogenate was centrifugated at 12000 g for 15 min at 4 °C. Total glutathione was measured in a reaction mixture consisting of 200 μl extract, 200 μl reagent solution A (110 mM Na_2_PO_4_, 40 mM NaH_2_PO_4_, 15 mM EDTA, 0.3 mM 5,5′-dithiobis-(2-nitrobenzoic acid)), and 160 μl reagent B (1 mM EDTA, 50 mM imidazole, and 1.5 units GR activity). 80 μl of NADPH was added to start the reaction. The change in absorbance was monitored at 412 nm. For GSSG, 200 μl extract was incubated at 25 °C for 1 h with 40 μl 2-vinylpyridine before measurement according to the method mentioned above. Similarly, GSH was estimated as the difference between total glutathione and GSSG.

### Metabolite extraction, separation, and quantification

The extraction procedure of metabolites was conducted according to methods of Roessner *et al*.^91^ and Rizhsky *et al*.^92^. Leaf samples were collected form 35 d of treatments and lyophilized in a FreeZone 4.5 system (Labconco, Kansas City, MO) until consistent weight. The lyophilized samples were then ground to a fine powder. Leaf tissue powder (20 mg) was transferred into a 10 ml centrifuge tube, and extracted in 1.4 ml of 80% (v/v) aqueous methanol at 200 rpm for 2 h. A 10 μl ribitol solution (2 mg ml^−1^) was added to tube as an internal standard prior to incubation. The samples were incubated in a water bath at 70 °C for 15 min. After centrifuged at 12000 rpm for 30 min, the supernatant was pipetted into new tube, and then 1.4 ml of water and 0.75 ml of chloroform were added in tube. The mixture was thoroughly vortexed and centrifuged for 5 min at 5000 rpm. 2 ml of the polar phase (methanol/water) was decanted into 1.5 ml HPLC vials and dried in a centrivap benchtop centrifugal concentrator (Labconco, Kansas City, MO). The dried polar phase was methoximated with 80 μl of methoxyamine hydrochloride (20 mg ml^−1^) at 30 °C for 90 min and was trimethylsilylated with 80 μl N-methyl-N-(trimethylsily)trifluoroacetamide (MSTFA) including 1% trimethylchlorosilane (TMCS) for 60 min at 70 °C.

The analysis procedure of GC-MS was followed by the method of Qiu *et al*.^93^. The extracts were analyzed with a PerkinElmer gas chromatograph coupled with a TurboMass-Autosystem XL mass spectrometer (PerkinElmer Inc., Waltham, MS). Equal extract (1 μl) was injected into a DB-5MS capillary column (30 m × 0.25 mm × 0.25 μm, Agilent J&W Scientific, Folsom, CA). The inlet temperature was maintained at 260 °C. Initial GC oven temperature was set at 80 °C after a 5 min solvent delay; the GC oven temperature was raised to 280 °C with 5 °C min^−1^ after injection for 2 min, and finally held at 280 °C for 15 min. For the injection temperature, it was set to 280 °C, and then the ion source temperature was adjusted to 200 °C. Helium was used as the carrier gas with a constant flow rate set at 1 ml min^−1^. The measurements were made with electron impact ionization (70 eV) in the full scan mode (m/z 30–550). The metabolites were identified by using TURBOMASS 4.1.1 software (PerkinElmer Inc.) coupled with commercially available compound libraries: NIST 2005 (PerkinElmer Inc.,Waltham, MS), Wiley 7.0 (John Wiley & Sons Ltd., Hoboken, NJ).

### Statistical analysis

The general linear model procedure for the analysis of variance (SAS 9.1, SAS Institute, Cary, NC) was used to determine the significance of main treatment effects and interaction of GABA and heat stress for all measured parameters. The significance of differences was tested using Fisher’s protected least significance test (LSD) with p = 0.05 at a given day of stress treatment.

## Additional Information

**How to cite this article**: Li, Z. *et al*. Metabolic pathways regulated by γ-aminobutyric acid (GABA) contributing to heat tolerance in creeping bentgrass (*Agrostis stolonifera*). *Sci. Rep*. **6**, 30338; doi: 10.1038/srep30338 (2016).

## Figures and Tables

**Table 1:** Derivitization, relative retention times, and mass to charge (m/z) ratios used for identification and quantification of the 57 metabolites identified in creeping bentgrass.

No.	RT (min)	Metabolites	m/z	No.	RT (min)	Metabolites	m/z
1	5.735	Glycine	58	30	16.201	D–Psicofuranose	245
2	6.758	Lactic Acid	117	31	16.445	Shikimic acid	204
3	6.996	Glycolic acid	147	32	16.555	Citric acid	233
4	7.170	Pyruvic acid	147	33	16.967	Quininic acid	73
5	7.402	Alanine	116	34	17.096	Fructose	103
6	7.962	Oxalic acid	220	35	17.321	Galactose	179
7	8.477	Phosphoric acid	211	36	17.366	Glucose	233
8	8.882	Malonic acid	233	37	17.565	Talose	233
9	9.056	Valine	144	38	17.701	Mannitol	205
10	9.655	Serine	132	39	18.531	Gluconic acid	205
11	9.867	Glycerol	103	40	18.898	Palmitic Acid	185
12	10.176	Threonine	130	41	19.284	Myo–Inositol	217
13	10.247	Proline	142	42	19.432	Allose	158
14	10.459	Succinic acid	147	43	19.735	Cinnamic acid	233
15	10.653	Glyceric acid	189	44	20.481	Linolenic acid	108
16	10.839	Itaconic acid	147	45	20.700	Stearic acid	185
17	12.468	Erythrulose	103	46	21.221	Glyceryl–glycoside	73
18	12.738	Malic acid	233	47	21.279	Mannose	211
19	13.137	Aspartic acid	233	48	21.672	D–Glycero–D–gulo–Heptose	185
20	13.182	5–Oxoproline	156	49	21.724	Galacturonic acid	233
21	13.311	GABA	158	50	22.721	Maltose	204
22	13.600	Threonic acid	245	51	24.453	Turanose	233
23	14.347	Glutamic acid	128	52	25.502	Cellobiose	222
24	14.978	Xylose	233	53	26.506	Galactinol	204
25	15.306	Levoglucosan	204	54	27.607	Dulcitol	245
26	15.441	Fucose	233	55	27.825	Gentiobiose	204
27	15.821	Aconitic acid	211	56	30.799	β–Sitosterol	185
28	15.950	Ribonic acid	189	57	31.494	Sucrose	70
29	16.001	Lyxofuranose	233				
